# Prognostic role of serum p53 antibodies in lung cancer

**DOI:** 10.1186/s12885-015-1174-4

**Published:** 2015-03-18

**Authors:** Manlio Mattioni, Silvia Soddu, Andrea Prodosmo, Paolo Visca, Salvatore Conti, Gabriele Alessandrini, Francesco Facciolo, Lidia Strigari

**Affiliations:** 1 Department of Experimental Oncology, Regina Elena National Cancer Institute, via Elio Chianesi 53, 00144 Rome, Italy; 2 Molecular Oncogenesis Laboratory, Department of Experimental Oncology, Regina Elena National Cancer Institute, via Elio Chianesi 53, 00144 Rome, Italy; 3 Pathology Department, Regina Elena National Cancer Institute, via Elio Chianesi 53, 00144 Rome, Italy; 4 Thoracic Surgery Unit, Regina Elena National Cancer Institute, via Elio Chianesi 53, 00144 Rome, Italy; 5 Laboratory of Medical Physic and Expert System, Regina Elena National Cancer Institute, via Elio Chianesi 53, 00144 Rome, Italy

**Keywords:** Lung cancer, Prognosis, *TP53* Gene mutations, p53 Protein expression, Serum p53 antibodies

## Abstract

**Background:**

Mutations in the *TP53* (Tumour Protein 53) gene can lead to expression of mutant p53 proteins that accumulate in cancer cells and can induce circulating p53 antibodies in cancer patients. Our aim was to evaluate the presence and prognostic role of these antibodies in lung cancer patients and to investigate whether they were related to p53 expression or *TP53* mutations in tumour tissues.

**Methods:**

A total of 201 lung cancer patients were evaluated for p53 antibodies by ELISA (Enzyme-Linked Immunosorbent Assay) and control was obtained from 54 patients with non-malignant disorders; p53 expression was evaluated in 131 of the lung cancer patients by immunohistochemistry and *TP53* mutations were then investigated in 53 tumours positively staining for p53 and in 12 tumours without p53 overexpression, whose DNA was available for direct sequencing.

**Results:**

Our results show that 20.4% of cancer patients have positive levels of p53 antibodies, while none of the controls resulted positive. High levels of p53 expression are detected in 57.3% of cases and a significant correlation between serum p53 antibodies and high levels of p53 expression in the corresponding tumours is observed. In non-small cell lung cancer, p53 antibodies are significantly associated with poorly differentiated tumours; furthermore, high levels of p53 expression significantly correlated with squamous cell carcinoma and tumours with highest grade. Survival time of non-small cell lung cancer patients low/negative for serum p53 antibodies was significantly longer compared to patients with positive levels (p = 0.049); in particular, patients with squamous cell carcinoma, but not adenocarcinoma, low/negative for these antibodies show a significant better survival compared to serum-positive patients (p = 0.044).

**Conclusions:**

In our study, detection of serum p53 antibodies in non-small cell lung cancer patients has been shown to be useful in identifying subsets of patients with poor prognosis. A significant correlation between the presence of serum p53 antibodies in lung cancer patients and p53 overexpression in the corresponding tumours was also observed. We did not find a significant correlation between levels of serum p53 antibodies and *TP53* mutations in the corresponding tumours.

## Background

Lung cancer represents the most common cancer in developed countries and the leading cause of tumour death in the world [1]. Usually, lung cancer does not show symptoms in early stages and most patients are diagnosed in advanced stages, when they are inoperable; therefore, the search for reliable diagnostic or prognostic biomarkers may be of remarkable clinical importance. The tumour suppressor p53 is involved in cell growth regulation, cell cycle progression, DNA repair and apoptosis; mutations in the *TP53* gene, the most common genetic alterations in human cancers, can lead to production of dysfunctional p53 proteins that may allow the survival of genetically unstable cells that can turn into malignant cells [2]. Mutant p53 proteins show a longer half-life than wild-type p53, resulting in accumulation in cancer cells; p53 overexpression can in turn induce circulating p53 antibodies (p53Abs) in patients bearing various types of cancer, including lung cancer, presumably because the altered conformation of p53 produced by mutations may trigger an autoimmune response once the protein has been released from tumour cells [3]. There is a close correlation between serum p53Abs and p53 overexpression in tumour tissues, thus p53Abs can be considered as markers for the presence of *TP53* mutations [4]. In lung cancer, *TP53* mutations arise early and p53 overexpression was detected in pre-neoplastic lesions, such as bronchial dysplasia. In addition, serum p53Abs were found in heavy smokers several months before the diagnosis of lung cancer [5].

In a systematic review of published studies, the frequency of serum p53Abs in most of cancer patients resulted higher than in healthy and benign controls; therefore, detection of serum p53Abs may have potential diagnostic value for different types of cancer, including lung cancer [6]. However, another meta-analysis suggested that the low sensitivity of serum p53Abs limited their use in the screening of lung cancer [7]. A combination of serum p53Abs with other conventional markers increased the sensitivity and specificity for detecting lung cancer [8]. Serum p53Abs may be useful also for predicting chemosensitivity in lung cancer: actually, serum p53Ab levels significantly decreased after neoadjuvant chemotherapy and low levels of serum p53Abs before neoadjuvant chemotherapy correlated with high objective chemoresponse rate [9].

Prognostic implications in lung cancer of p53Abs are controversial: in non-small cell lung cancer (NSCLC), p53Abs were found to be related to short survival, but some studies showed the absence of correlation; in small cell lung cancer (SCLC), either a better survival in patients with high levels of p53Abs or a shorter survival in p53Ab positive patients with limited disease, as well as lack of prognostic relevance have been observed [10]. The reported differences in prognostic correlations may be partially due to the different sensitivity and reactivity of the methods employed or to the peculiar characteristics of each investigated population.

Furthermore, as results of most studies are limited to the prognostic role of serum p53Abs, the aim of our work was not only to determine serum p53Abs in lung cancer patients and evaluate their prognostic role, but also to examine whether these antibodies were associated with p53 protein expression or mutations in corresponding tumour tissues, as p53 overexpression is believed to be an important trigger for the production of serum p53Abs. For these purposes, we simultaneously determined in lung cancer patients serum p53Abs by a highly specific ELISA, p53 protein expression by immunohistochemistry (IHC) and *TP53* gene mutations by direct sequencing of exons 2–11, in corresponding tumours.

## Methods

### Patients

A total of 255 patients were evaluated (Table 1). Of these patients, 201 had histologically diagnosed primary lung cancer: 13 patients had SCLC and 188 patients had NSCLC. Control was obtained from 54 patients with benign lung diseases or disorders not related to respiratory diseases. All patients were enrolled at the Thoracic Surgery Unit of the Regina Elena National Cancer Institute, Rome, Italy, between June 2004 and August 2006.

**Table 1:** **Patient characteristics**

	*Lung cancer patients*	*Controls*
**Age** (years)		
≤60	47 (23.4%)	27 (50.0%)
>60	154 (76.6%)	27 (50.0%)
Total	201	54
**Sex**		
Male	157 (78.1%)	27 (50.0%)
Female	44 (21.9%)	27 (50.0%)
Total	201	54
**Histological type**		
NSCLC		
*Adenocarcinoma*	79 (39.3%)	
*Squamous cell carcinoma*	70 (34.8%)	
*Bronchioloalveolar carcinoma*	6 (3.0%)	
*Large cell carcinoma*	5 (2.5%)	
*Anaplastic carcinoma*	19 (9.4%)	
*Mixed carcinoma*	9 (4.5%)	
SCLC	13 (6.5%)	
Total	201	
**NSCLC stage**		
I	68 (36.2%)	
II	24 (12.8%)	
III	40 (21.3%)	
IV	45 (23.9%)	
Missing	11 (5.9%)	
Total	188	
**NSCLC grade**		
G1	4 (2.1%)	
G2	57 (30.3%)	
G3	72 (38.3%)	
Missing	55 (29.3%)	
Total	188	

NSCLC was pathologically staged according to the TNM (tumour-nodes-metastasis) classification (Union International Contre le Cancer 2002): stage IA (n. 32); stage IB (n. 36); stage IIA (n. 1); stage IIB (n. 23); stage IIIA (n. 32); stage IIIB (n. 8); stage IV (n. 45). Most patients with I to IIIA stages underwent surgery for their primary tumours at the Thoracic Surgery Unit of the Regina Elena National Cancer Institute, Rome, Italy, while most patients with stages IIIB and IV had chemotherapy. Radiotherapy was added if necessary. NSCLC grade was expressed as G parameter, universally accepted as a pathological descriptor for cancer: the more the tumour sections resemble the original normal tissue, when observed by pathologist, the lower the grade, ranging from G1 (well differentiated) to G3 (poorly differentiated), being G2 an intermediated form (moderately differentiated).

The study was approved by the Ethical Committee on human experimentation of the promoting institution, Regina Elena National Cancer Institute, Rome, Italy. All participants in the study gave their written informed consent. The lung cancer patients were followed until October 2009.

### Serum p53Ab assay

Serum samples were obtained from patients at the time of diagnosis. All samples were aliquoted, coded, and stored at −80°C until assays were performed. p53Abs were detected by a commercially available, highly specific ELISA kit (Anti-p53 ELISA II kit, PharmaCell, Paris, France), using micro-titre plates coated either with human recombinant p53 protein to detect specific p53Abs or with control proteins to reveal non-specific interactions. The assay was performed according to the manufacturer’s instructions. All samples were tested blindly, twice in the same assay. Absorbance was measured at 450 nm and 620 nm, using a programmable ELISA reader. p53Ab levels ≥1.2 units/ml were considered as positive, according to the manufacturer’s suggestion; this cut-off was in agreement with other studies [11]. p53Ab levels <1.2 units/ml were considered as low/negative.

### p53 protein expression

**Figure 1:**
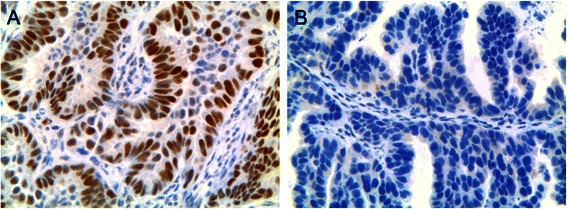
**Immunohistochemistry of p53 in a lung adenocarcinoma. A)** p53 positive: strong p53 immunoreactivity of the nuclei. **B)** p53 negative: no p53 nuclear staining. Magnification ×20.

p53 immunostaining was performed by using the monoclonal antibody D07 (Novocastra, Menarini, Florence, Italy). Immunoreactions were revealed by a streptavidin-biotin enhanced immunoperoxidase technique (Super Sensitive MultiLink, Menarini) in an automated autostainer (Vysion Biosystems Bond; Menarini, Florence, Italy). Diaminobenzidine was used as the chromogenic substrate. Tumour samples with >10% of tumour cells showing positive nuclear staining were considered as positive (Figure 1).

### Genomic DNA extraction, PCR (Polymerase Chain Reaction) amplification of the *TP53* exons 2–11, and mutational analysis by direct sequencing

DNA extraction from formalin-fixed paraffin-embedded (FFPE) tissue samples on histological sections was performed. For microdissection, appropriate tissue blocks were selected and multiple serial sections were stained with haematoxylin and eosin, and visualized with an inverted microscope. Under an operating microscope, tumour epithelium tissues were dissected from the corresponding unstained 10-μm sections by using a sterilized needle. These tissues were collected, deparaffinised and digested with proteinase K overnight in lysis buffer, before DNA isolation with the QIAamp DNA FFPE Tissue Kit (QIAGEN, USA). DNA was eluted in a volume of 50–150 μl of water. DNA concentrations were measured by Nanodrop and the amount of isolated DNA ranged from 50–300 ng/μl. About 50–100 ng of genomic DNA was used for eleven PCR reactions to amplify the regions of the *TP53* exons 2–11. Primers were designed to amplify these DNA regions (Table 2). All PCR reactions were in a volume of 20 μl containing 8 pmol of each primer, 0.20 mM each dNTP, 1-X of Kapa Taq Buffer A with MgCl_2_, 0.25 units of Kapa Taq (Kapa Biosystems, South Africa), and the appropriate volume of H_2_O. Thermocycle PCR protocol was performed as follows: 2 minutes at 95°C, 35–40 cycles at 95°C for 30 seconds, 58°C for 30 seconds, and 72°C for 30 seconds, followed by 72°C for 2 minutes. PCR amplicons obtained from DNA were purified by gel filtration with Sephadex G-100 (GE Healthcare, USA). About 15–30 ng of PCR products were sequenced directly by using the Big Dye V3.1 Cycle-Sequencing kit (Applied Biosystems, USA) with proper primers (Table 2). After sequencing reaction, the entire reactions of every mixture were purified by gel filtration with Sephadex G-50 (GE Healthcare, USA) and analyzed by 3730 DNA Analyzer (Applied Biosystems, USA).

**Table 2:** **Primers for** ***TP53*** **exon 2–11 amplification and sequencing analysis**

***TP53*** **exon 2**	Fw	5′ GGTTGGAAGTGTCTCATGC 3′
	Rv	5′ TTCGCTTCCCACAGGTCTC 3′*
***TP53*** **exon 3**	Fw	5′ AGAGACCTGTGGGAAGCGA 3′*
	Rv	5′ GCAGTCAGAGGACCAGGT 3′
***TP53*** **exon 4a**	Fw	5′ TGGTCCTCTGACTGCTCTT 3′
	Rv	5′ AAGAAGCCCAGACGGAAACC 3′*
***TP53*** **exon 4b**	Fw	5′ AGCTCCCAGAATGCCAGAG 3′*
	Rv	5′ CAGGCATTGAAGTCTCATGG 3′
***TP53*** **exon 5**	Fw	5′GCCCTGACTTTCAACTCTG 3′
	Rv	5′ GGCAACCAGCCCTGTCGT 3′*
***TP53*** **exon 6**	Fw	5′ CAGGCCTCTGATTCCTCAC 3′
	Rv	5′ CTACTGCTCACCTGGAGG 3′*
***TP53*** **exon 7**	Fw	5′ CTTGGGCCTGTGTTATCTC 3′
	Rv	5′AAATCGGTAAGAGGTGGGC 3′*
***TP53*** **exon 8**	Fw	5′ ACAAGGGTGGTTGGGAGTAG 3′*
	Rv	5′ CCTCCACCGCTTCTTGTCC 3′
***TP53*** **exon 9**	Fw	5′CAAGAAGCGGTGGAGGAGA 3′*
	Rv	5′ TCCACTTGATAAGAGGTCCCA 3′
***TP53*** **exon 10**	Fw	5′ CCATCTTTTAACTCAGGTACTG 3′
	Rv	5′TGAAGGCAGGATGAGAATGG 3′*
***TP53*** **exon 11**	Fw	5′CACAGACCCTCTCACTCAT 3′
	Rv	5′TCCTGGGTGCTTCTGACG 3′*

*Primers used for sequencing; Fw, forward; Rv, reverse.

### Statistical analysis

All statistical analyses were performed by using R [12]. For all measured endpoints, all times were calculated after primary diagnosis of lung cancer and patients were censored at the time of the specific event. Actuarial curves of the length of time until the death were calculated by the Kaplan and Meier product-limit method. The comparison of the actuarial curves was evaluated by the log-rank test. Tests for statistical significance were performed with the chi-square and *t*-test for categorical and continuous variables, respectively. Multivariate analysis of prognostic factors was performed using the Cox proportional hazard model. In both univariate and multivariate analysis, p-values of <0.05 were considered statistically significant.

## Results

### Serum p53Abs in lung cancer patients

Sera from 201 patients with primary lung carcinoma were evaluated for the presence of p53Abs using a highly specific ELISA kit. Our results demonstrated that 20.4% of lung cancer patients (41 out of 201) had positive levels of serum p53Abs, while none of the 54 controls resulted positive (Table 3). Our results agree with data from other studies on lung carcinoma where the frequency of p53Abs in serum ranged from 18.8% to 32.1% of the patients [13,14]. In particular, 36 out of 188 NSCLC patients (19.1%) and 5 out of 13 SCLC patients (38.5%) were positive for serum p53Abs.

**Table 3:** **Serum p53Abs in lung cancer patients and controls, p53 content in lung cancer tissues**

	No. of cases	No. of positive	No. of low/negative
Lung cancer sera	201	41^a^ (20.4%)	160^a^ (79.6%)
Control sera	54	0^a^	54^a^
Lung cancer tissues	131	75^b^ (57.3%)	56^b^ (42.7%)

^a^p53Ab, ^b^p53 protein expression.

### Serum p53Abs and p53 tumour expression

High levels of p53 tumour expression were demonstrated in 75 out of 131 lung cancer patients (57.3%), for whom tumour tissues were available (Table 3). Among these patients, 19 (16 NSCLC and 3 SCLC) out of 22 positive for serum p53Abs resulted also positive for p53 tumour overexpression (86.4%), while 56 (53 NSCLC and 3 SCLC) out of 109 low/negative for serum p53Abs were positive for p53 tumour overexpression (51.4%). A significant correlation (p = 0.005) between positive levels of circulating p53Abs and high levels of p53 expression in the corresponding tumours was observed (Table 4).

**Table 4:** **Relationship between serum p53Abs and p53 tumour staining in lung cancer patients**

p53 tumour staining	No. of p53Ab-positive	No. of p53Ab- low/negative	p-value
Positive	19 (86.4%)	56 (51.4%)	0.005
Negative	3 (13.6%)	53 (48.6%)	

### Serum p53Abs, p53 tumour expression and clinicopathologic parameters

The relationships among levels of p53Abs or p53 accumulation and clinical or tumour biological parameters of lung cancer patients were evaluated. No difference was found with regard to age and sex. By histological types, positive levels of p53Abs were more frequently found in patients with squamous cell carcinoma than adenocarcinoma (p = 0.068), while the numbers of other subgroups were too few to draw meaningful conclusions (Table 5). In NSCLC, there was a difference of borderline significance (p = 0.054) in the incidence of p53Abs between stage I and the more advanced stages II-IV. Finally, in NSCLC the presence of p53Abs was significantly correlated with tumours showing poorly differentiated grade (p = 0.016). Furthermore, a significant correlation between high levels of p53 protein expression and squamous cell carcinoma compared to adenocarcinoma was observed (p = 0.008), while no difference was found with regard to NSCLC stage (Table 6); p53 overexpression was also significantly associated with NSCLC of highest grade (p = 0.012).

**Table 5:** **Correlation between serum p53Abs and clinicopathologic features**

	*No. of cases*	*No. of p53Ab-positive*
**Age** (years)		
≤60	47	11 (23.4%)
>60	154	30 (19.5%)
**Sex**		
Male	157	29 (18.5%)
Female	44	12 (27.3%)
**Histological type**		
NSCLC		
*Adenocarcinoma*	79	10 (12.7%)
*Squamous cell carcinoma*	70	18 (25.7%)
*Bronchioloalveolar carcinoma*	6	0 (0%)
*Large cell carcinoma*	5	1 (20.0%)
*Anaplastic carcinoma*	19	5 (26.3%)
*Mixed carcinoma*	9	2 (22.2%)
SCLC	13	5 (38.5%)
**NSCLC stage**		
I	68	7 (10.3%)
II	24	7 (29.2%)
III	40	9 (22.5%)
IV	45	9 (20.0%)
Missing	11	4 (36.4%)
Total	188	36 (19.1%)
II-IV	109	25 (22.9%)
**NSCLC grade**		
G1	4	0 (0%)
G2	57	4 (7.0%)
G3	72	18 (25.0%)
Missing	55	14 (25.5%)
Total	188	36 (19.1%)

**Table 6:** **Relationship between p53 overexpression and clinicopathologic parameters**

	*No. of cases*	*No. of patients with p53 overexpression*
**Age** (years)		
≤60	27	16 (59.3%)
>60	104	59 (56.7%)
Total	131	75 (57.3%)
**Sex**		
Male	101	57 (56.4%)
Female	30	18 (60.0%)
Total	131	75 (57.3%)
**Histological type**		
NSCLC		
*Adenocarcinoma*	59	26 (44.1%)
*Squamous cell carcinoma*	46	33 (71.7%)
*Bronchioloalveolar carcinoma*	5	1 (20.0%)
*Large cell carcinoma*	4	4 (100%)
*Anaplastic carcinoma*	4	4 (100%)
*Mixed carcinoma*	5	1 (20.0%)
SCLC	8	6 (75.0%)
Total	131	75 (57.3%)
**NSCLC stage**		
I	62	35 (56.4%)
II	17	9 (52.9%)
III	22	15 (68.2%)
IV	18	8 (44.4%)
Missing	4	2 (50.0%)
Total	123	69 (56.1%)
**NSCLC grade**		
G1	3	1 (33.3%)
G2	53	21 (39.6%)
G3	60	40 (66.7%)
Missing	7	7 (100%)
Total	123	69 (56.1%)

### *TP53* gene mutations and clinicopathologic features

**Figure 2:**
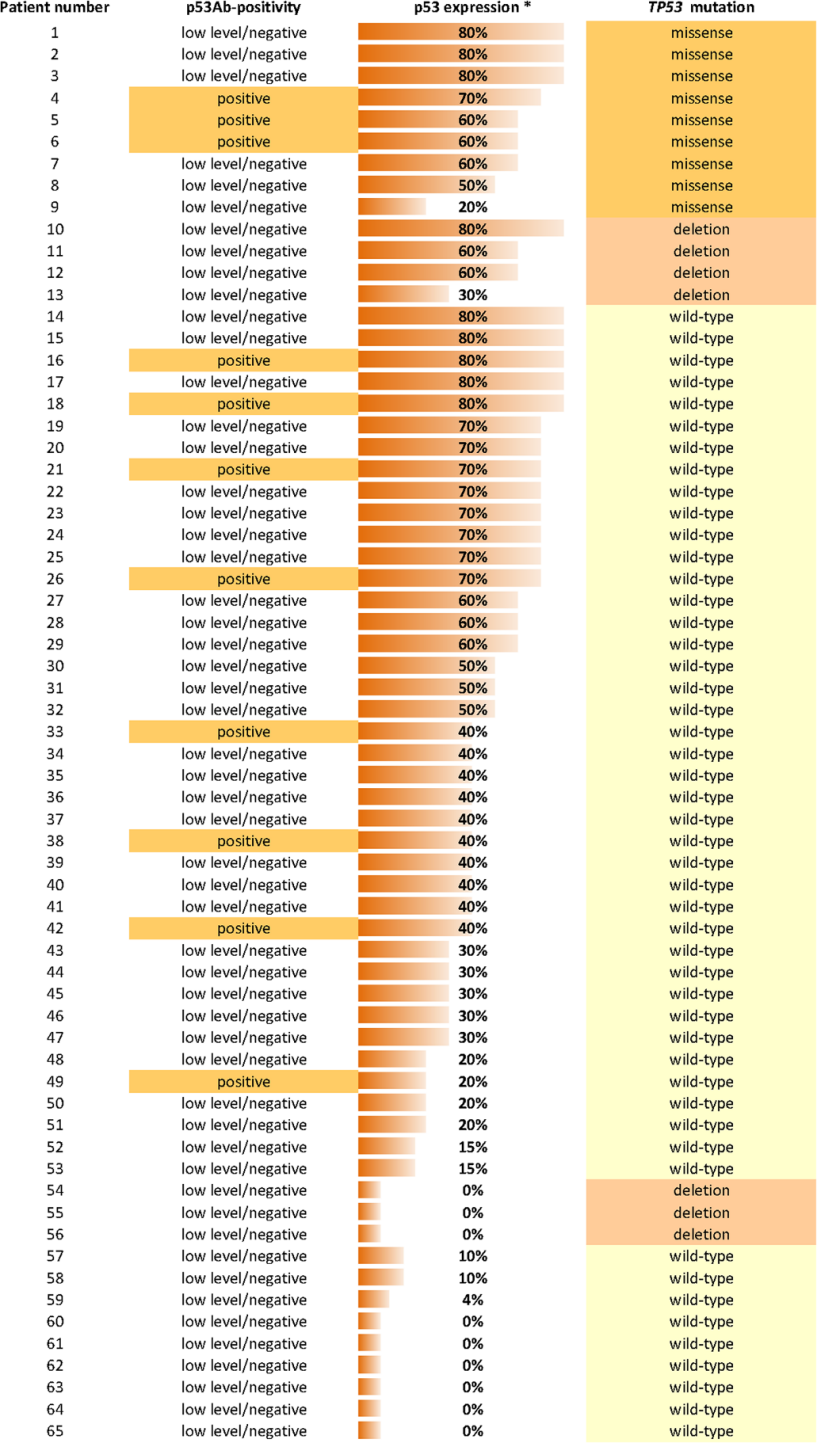
**Overlap between p53Ab-positivity, p53 expression and*TP53*mutation in lung cancer patients (n = 65).** * Percentage of tumour cells showing p53 staining.

From the 75 tumours positively staining for p53 protein, only 53 had enough tumour tissue to be investigated for *TP53* gene mutations in exons 2–11. Of these 53 tumour samples, 13 (all NSCLC, 24.5%) showed *TP53* mutations (9 missense mutations and 4 deletions), while 40 (39 NSCLC and 1 SCLC, 75.5%) showed no mutation; the median rate of tumour cells with p53 overexpression in tumours with mutations was 60%, while in tumours without mutations was 40% (p = 0.04). Mutational analysis was also performed in 12 patients without p53 protein overexpression by IHC (all NSCLC), whose DNA was available for direct sequencing; in this group, compatible with p53 negative staining, we found 9 patients with wild-type p53 and 3 patients with p53 deletions (Figure 2).

The most frequent region of mutation was exon 5 (6/16), followed by exon 8 (5/16), exon 4 and exon 2 (2/16); only one mutation was found in exon 6 (Table 7). Of the 16 mutations, 9 (56.2%) were missense mutations and 7 (43.7%) were deletions; 5 out of 9 point mutations (55.6%) were base pair transversions (G = > T 4/9, G = > C 1/9), while 4 (44.4%) were transitions (C = > T). The same mutation p.Pro12Leu was found in exon 2 in two patients; according to both the IARC (International Agency for Research on Cancer) *TP53* mutational database and UMD (Universal Mutation Database) *TP53* mutation database, this mutation is functional in yeast but extremely rare. Thus, to verify its presence in two different patients, we performed two independent DNA purifications, PCR reactions, and direct sequences for each biopsy. The results confirmed the presence of the same mutation in the two patients, suggesting that its rarity might be due, at least in part, to the fact that many studies are limited to exons 5–9, where *TP53* mutations are more frequent.

**Table 7:** **Details of lung cancer patients with** ***TP53*** **mutations in their tumours**

Patient number	Sex	Age	Histology*	TNM	G^§^	p53Abs	IHC	Exon	Codon	Nucleic acid change	Aminoacid change
8	M	70	SCC	IB	3	N&	P^#^	2	12	c.35C > T	Pro12Leu
4	M	70	ADC	IB	3	P	P	2	12	c.35C > T	Pro12Leu
54	M	59	ADC	IIB	3	N	N	4	66-81	c.196_241del46	Frameshift
1	M	72	SCC	IA	3	N	P	4	110	c.329G > T	Arg110Leu
55	M	77	SCC	IA	3	N	N	5	152	c.456delG	Frameshift
10	F	55	SCC	IIB	3	N	P	5	154	c.460_474del15	Frameshift
2	M	65	LCC	IB	3	N	P	5	157	c.469G > T	Val157Phe
5	M	41	APC	IIB	3	P	P	5	158	c.473G > T	Arg158Leu
3	M	76	SCC	IIB	3	N	P	5	159	c.475G > C	Ala159Pro
7	M	73	SCC	IB	2	N	P	5	167	c.501G > T	Gln167His
11	F	68	ADC	IB	2	N	P	6	199-200	c.596_598delGAA	Frameshift
56	M	70	ADC	IA	2	N	N	8	269	c.807delC	Frameshift
6	F	82	ADC	IIB	2	P	P	8	273	c.817C > T	Arg273Cys
9	M	50	ADC	IB	3	N	P	8	282	c.844C > T	Arg282Trp
12	F	70	SCC	IB	2	N	P	8	288	c.862delA	Frameshift
13	M	79	SCC	IIB	3	N	P	8	288	c.862delA	Frameshift

*SCC, squamous cell carcinoma; ADC, adenocarcinoma; APC, anaplastic carcinoma; LCC, large cell carcinoma. ^§^Grade; &Low/Negative, ^#^Positive.

In our cohort, *TP53* mutations were more frequently associated with NSCLC of highest grade, although the difference did not reach a statistical significance, likely due to the small size of the sample. Other clinicopathologic features, such as age and sex of patients, histological type of tumours and NSCLC stage were not significantly correlated with *TP53* mutations (Table 8).

**Table 8:** **Comparison of** ***TP53*** **mutations and clinicopathologic features**

	*No. of cases*	*No. of patients with TP53 mutations*
**Age** (years)		
≤60	12	4 (33.3%)
>60	53	12 (22.7%)
Total	65	16 (24.6%)
**Sex**		
Male	51	12 (23.5%)
Female	14	4 (28.6%)
Total	65	16 (24.6%)
**Histologic type**		
NSCLC		
*Adenocarcinoma*	27	6 (22.2%)
*Squamous cell carcinoma*	27	8 (29.6%)
*Bronchioloalveolar carcinoma*	2	0 (0%)
*Large cell carcinoma*	4	1 (25.0%)
*Anaplastic carcinoma*	2	1 (50.0%)
*Mixed carcinoma*	2	0 (0%)
SCLC	1	0 (0%)
Total	65	16 (24.6%)
**NSCLC stage**		
I	41	10 (24.4%)
II	9	6 (66.7%)
III	10	0 (0%)
IV	4	0 (0%)
Total	64	16 (25.0%)
**NSCLC grade**		
G1-G2	28	5 (17.9%)
G3	35	11 (31.4%)
Missing	1	0 (0%)
Total	64	16 (25.0%)

### Serum p53Abs and *TP53* gene mutations

The corresponding sera of the 53 patients with tumours positively staining for p53 protein, investigated for *TP53* mutations, were available. Eleven of these patients (all with NSCLC) were positive for serum p53Abs (20.8%) and three showed missense mutations in the corresponding tumours (27.3%), while ten patients (all with NSCLC) out of the 42 with low/negative-serum (41 with NSCLC and 1 with SCLC) had missense mutations or deletions in their tumours (23.8%). The sera of the 12 patients without p53 protein overexpression, whose DNA was available for *TP53* mutational analysis, were all low/negative for serum p53Abs (Figure 2). In our limited cohort, there was not a statistically significant association between serum p53Abs and the presence of *TP53* mutations in the corresponding tumours.

### Serum p53Abs and clinical outcome

**Figure 3:**
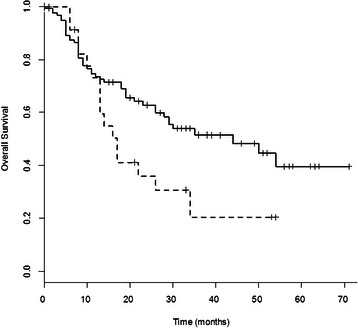
**Survival curves of NSCLC patients with p53Ab-positive or p53Ab-low/negative- serum.** Survival curves of NSCLC patients with p53Ab-positive-serum (dotted line, n = 36) or p53Ab-low/negative-serum (unbroken line, n = 152). Patients with p53Ab-low/negative-serum had a significantly longer overall survival than those with p53Ab-positive-serum (p = 0.049).

**Figure 4:**
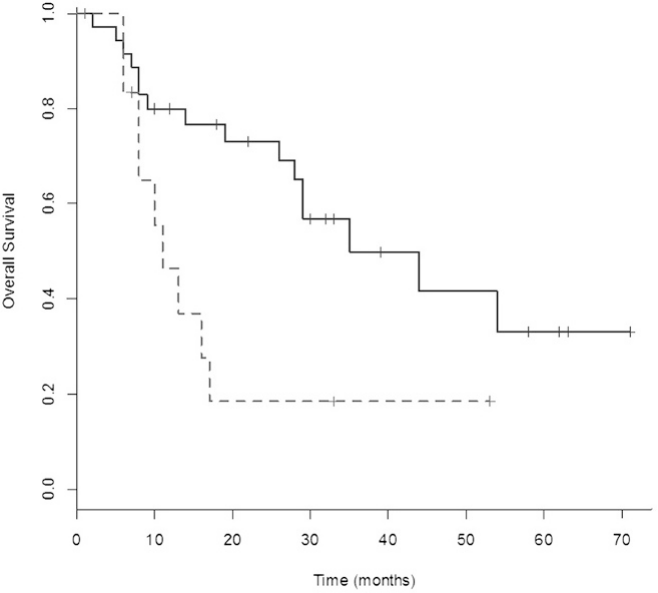
**Survival curves of squamous cell carcinoma patients with p53Ab-positive or p53Ab-low/negative-serum.** Survival curves of patients with squamous cell carcinoma of the lung with p53Ab-positive-serum (dotted line, n = 18) or p53Ab-low/negative-serum (unbroken line, n = 52). Patients with p53Ab-low/negative-serum showed a significantly longer overall survival than those with p53Ab-positive- serum (p = 0.044).

**Figure 5:**
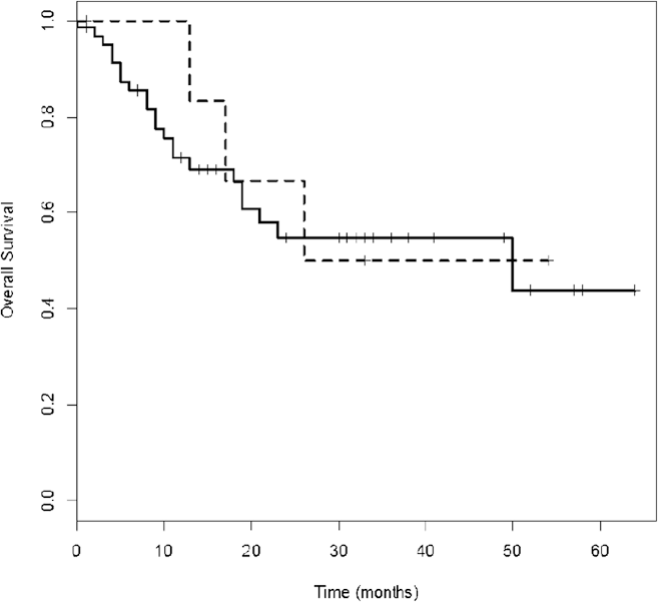
**Survival curves of adenocarcinoma patients with p53Ab-positive or p53Ab-low/negative-serum.** Survival curves of patients with adenocarcinoma of the lung with p53Ab-positive-serum (dotted line, n = 10) or p53Ab-low/negative-serum (unbroken line, n = 69). No difference in overall survival was observed between the two groups (p = 0.973).

We then considered the correlation between the presence of p53Abs in the serum and survival. The overall survival time of patients with p53Ab-low/negative-serum and NSCLC was significantly longer compared to serum-positive patients (p = 0.049); the rates of survival at 30 months were 54% versus 31% in patients with low/negative or positive-serum, respectively (Figure 3). In particular, patients with squamous cell carcinoma and p53Ab-low/negative-serum showed a statistically significant advantage in survival rate at 30 months compared to serum-positive patients, 57% versus 18% respectively (p = 0.044, Figure 4), while no difference was observed in patients with adenocarcinoma (Figure 5); similarly, no difference was found with regard to stage or grade. In this study, there was no correlation between p53 overexpression or *TP53* mutations in NSCLC and survival.

Multivariate analysis comprised age (as categorical, >60 years), sex, histological type, stage, grade, and serum p53Abs. Stage emerged as an independent prognostic factor (p < 0.001), while serum p53Abs resulted an independent variable as a trend (p = 0.096).

## Discussion

Our data showed that 20.4% of lung cancer patients had positive levels of serum p53Abs, while none of the controls showed positive levels of these antibodies. A significant correlation between positive levels of p53Abs and p53 overexpression in the corresponding tumours was observed (p = 0.005), suggesting that the presence of p53Abs is related to the accumulation of p53 protein in the primary tumour. In NSCLC, positive levels of p53Abs were associated with squamous cell carcinoma rather than adenocarcinoma (p = 0.068), a different frequency also previously reported [9]. In our study, p53Abs were correlated with II-IV stages compared to I stage (p = 0.054); similarly, the incidence of p53Abs was previously found higher in lung cancer patients with advanced stages III-IV compared to patients with early stages I-II [9]. We observed that the presence of p53Abs was significantly related to tumours with poorly differentiated grade (p = 0.016). In NSCLC, p53 overexpression significantly correlated with squamous cell carcinoma compared to adenocarcinoma (p = 0.008) and with tumours showing highest grade (p = 0.012). In previous studies, a statistically significant correlation between p53 overexpression and squamous cell carcinoma compared to adenocarcinoma was also reported and, in particular, Rybarova et al. found a correlation between p53 overexpression and poorly differentiated tumours in NSCLC [15,16]. Finally, we did not find a significant correlation between positive levels of serum p53Abs and *TP53* mutations in the corresponding tumours, nor between *TP53* mutations and clinicopathologic features, presumably due to the small number of suitable tumour samples, while a significant correlation between *TP53* mutations and tumours with the highest median rate of p53 overexpression was observed within the p53 protein-positive tumour samples (p = 0.04). In particular, *TP53* mutations were found in three out of 12 p53 protein-negative samples; the absence of p53 protein expression in these samples may be due to the type of mutations, as they were frameshift mutations. On the other hand, four samples with frameshift mutations showed p53 protein overexpression; in these samples, overexpression of wild-type p53 protein might be attributed to inhibition of MDM2 (Mouse Double Minute 2 homolog)-mediated degradation of p53 or activation of p14ARF (Alternate Reading Frame protein14), since p14ARF binds to MDM2, thus preventing MDM2 binding to p53 and subsequent p53 degradation [17].

In the present study, survival time of NSCLC patients low/negative for serum p53Abs was significantly longer than in positive patients (p = 0.049); moreover, patients with squamous cell carcinoma low/negative for serum p53Abs showed a significant increase in survival rate compared to positive patients (p = 0.044). Our results are in agreement with other studies, in which the presence of serum p53Abs has been associated with a poor prognosis. In a study by Mack et al., serum p53Abs were significantly correlated with a poor survival in NSCLC patients [18]. Laudanski et al. also showed a significant association between serum p53Abs and poor survival in NSCLC patients, even though in a multivariate analysis only the presence of *TP53* mutations remained an independent, significant unfavourable prognostic factor for survival [15]. Bergqvist et al., in a study regarding patients with advanced NSCLC, reported a significant poor survival in patients positive for serum p53Abs with adenocarcinoma, but not in positive patients with squamous cell carcinoma [19]. Recently, Hashim et al. found that in NSCLC the levels of serum p53Abs were significantly higher in stage IV compared to stages I-III [20]. However, some groups found no correlation with survival, while others showed a longer survival in lung cancer patients positive for serum p53Abs compared to negative patients, even though the difference did not reach a statistical significance [21,22].

The mechanisms for the relationship between serum p53Abs and prognosis are unclear. Usually, these antibodies are considered as markers of *TP53* mutations or p53 overexpression in tumours, thus reflecting a poor prognosis; however, the presence of serum p53Abs with a T-cell response may have a favourable impact on survival by destructing tumour cells expressing a dysfunctional p53. Serum p53Abs have been reported to be directed against both mutant and wild-type p53 proteins, where they recognize specific epitopes in the amino and carboxyl-termini, and the antigenic load due to p53 overexpression triggers the development of these antibodies [23]. However, only 20-40% of cancer patients with *TP53* mutations show circulating p53Abs, suggesting that other biological characteristics may be involved in this response, such as specific combination of Major Histocompatibility Complex classes I and II molecules expressed by each patient [3]. These observations may in part explain the reported differences in prognostic correlations of serum p53Abs.

Finally, in our study we did not find any significant correlation between p53 overexpression or *TP53* mutations and survival in NSCLC, likely because of the reduced number of suitable tumour samples compared to serum samples. In some studies, p53 accumulation and *TP53* mutations have been reported as markers of poor survival in NSCLC. Laudanski et al. showed that *TP53* mutations and p53 overexpression were significantly correlated with poor survival in NSCLC; however, in a multivariate analysis, only the presence of *TP53* mutations remained an independent, significant unfavourable prognostic factor for survival [15]. Tsao et al. found that patients with NSCLC overexpressing p53 showed significant shorter survival compared to patients with p53-negative tumours, while *TP53* mutations were not prognostic for survival [24]. However, other groups reported no correlation between p53 accumulation and survival in NSCLC or a not clear role for *TP53* mutations as a prognostic marker for survival in NSCLC [25,26].

## Conclusions

In our investigation the presence of serum p53Abs, but not p53 tumour overexpression or *TP53* mutations, identifies subsets of NSCLC patients with a shorter survival. Furthermore, positive levels of p53Abs were significantly correlated with p53 overexpression in the corresponding tumours, suggesting that the production of these antibodies may be related to the accumulation of p53 protein in the primary tumour. We were unable to find any significant correlation between positive levels of serum p53Abs and *TP53* mutations in the corresponding tumours.

## Abbreviations

*TP53*: Tumour protein 53
ELISA: Enzyme-linked immunosorbent assay
p53Abs: p53 Antibodies
NSCLC: Non-small cell lung cancer
SCLC: Small cell lung cancer
IHC: Immunohistochemistry
TNM: Tumour-nodes-metastasis
PCR: Polymerase chain reaction
FFPE: Formalin-fixed paraffin-embedded
IARC: International agency for research on cancer
UMD: Universal mutation database
MDM2: Mouse double minute 2 homolog
p14ARF: Alternate reading frame protein 14

## References

[1] World Cancer Report 2014*.* Cancer worldwide: pages 16-53. In: Stewart BW, Wild CP, editors. Lyon, France: International Agency for Research on Cancer; 2014.

[2] Hanahan D, Weinberg RA (2011). Hallmarks of cancer: the next generation. Cell.

[3] Soussi T (2000). p53 antibodies in the sera of patients with various types of cancer: a review. Cancer Res.

[4] Gao RJ, Bao HZ, Yang Q, Cong Q, Song JN, Wang L (2005). The presence of serum anti-p53 antibodies from patients with invasive ductal carcinoma of breast: correlation to other clinical and biological parameters. Breast Cancer Res Treat.

[5] Lubin R, Zalcman G, Bouchet L, Trédaniel J, Legros Y, Cazals D (1995). Serum p53 antibodies as early markers of lung cancer. Nat Med.

[6] Zhang J, Xu Z, Yu L, Chen M, Li K (2014). Assessment of the potential diagnostic value of serum p53 antibody for cancer: a meta-analysis. PLoS One.

[7] Lei Q-Q, Liu J-W, Zheng H (2013). Potential role of anti-p53 antibody in diagnosis of lung cancer: evidence from a bivariate meta-analysis. Eur Rev Med Pharmacol Sci.

[8] Park Y, Kim Y, Lee J-H, Lee EY, Kim H-S (2011). Usefulness of serum anti-p53 antibody assay for lung cancer diagnosis. Arch Pathol Lab Med.

[9] Yu D-H, Li J-H, Wang Y-C, Xu J-G, Pan P-T, Wang L (2011). Serum anti-p53 antibody detection in carcinomas and the predictive values of serum p53 antibodies, carcino-embryonic antigen and carbohydrate antigen 12–5 in the neoadjuvant chemotherapy treatment for III stage non-small cell lung cancer patients. Clin Chim Acta.

[10] Kumar S, Mohan A, Guleria R (2009). Prognostic implications of circulating anti-p53 antibodies in lung cancer - a review. Eur J Cancer Care.

[11] Cioffi M, Riegler G, Vietri MT, Pilla P, Caserta L, Carratù R (2004). Serum p53 antibodies in patients affected with ulcerative colitis. Inflamm Bowel Dis.

[12] R Development Core Team (2004). R: a language and environment for statistical computing. 3-900051-07-0.

[13] Sangrajrang S, Sornprom A, Chernrungroj G, Soussi T (2003). Serum p53 antibodies in patients with lung cancer: correlation with clinicopathologic features and smoking. Lung Cancer.

[14] Cioffi M, Vietri MT, Gazzerro P, Magnetta R, D’Auria A, Durante A (2001). Serum anti-p53 antibodies in lung cancer: comparison with established tumor markers. Lung Cancer.

[15] Laudanski J, Niklinska W, Burzykowski T, Chyczewski L, Niklinski J (2001). Prognostic significance of p53 and bcl-2 abnormalities in operable non small cell lung cancer. Eur Respir J.

[16] Rybárová S, Hodorová I, Muri J, Mihalik J, Adamkov M, Svajdler M (2011). Prognostic significance of p53 protein and X-ray repair cross-complementing protein 1 in non-small cell lung cancer. Tumori.

[17] Wang Y-C, Lin R-K, Tan Y-H, Chen J-T, Chen C-Y, Wang Y-C (2005). Wild-type p53 overexpression and its correlation with MDM2 and p14ARF alterations: an alternative pathway to non-small-cell lung cancer. J Clin Oncol.

[18] Mack U, Ukena D, Montenarh M, Sybrecht GW (2000). Serum anti-p53 antibodies in patients with lung cancer. Oncol Rep.

[19] Bergqvist M, Brattström D, Larsson A, Hesselius P, Brodin O, Wagenius G (2004). The role of circulating anti-p53 antibodies in patients with advanced non-small cell lung cancer and their correlation to clinical parameters and survival. BMC Cancer.

[20] Hashim M, Sayed M, Samy N, Elshazly S (2011). Prognostic significance of telomerase activity and some tumor markers in non-small cell lung cancer. Med Oncol.

[21] Bergqvist M, Brattström D, Lamberg K, Hesselius P, Wernlund J, Larsson A (2003). The presence of anti-p53 antibodies in sera prior to thoracic surgery in non small cell lung cancer patients: its implications on tumor volume, nodal involvement, and survival. Neoplasia.

[22] Neri M, Betta P, Marroni P, Filiberti R, Cafferata M, Mereu C (2003). Serum anti-p53 autoantibodies in pleural malignant mesothelioma, lung cancer and non-neoplastic lung diseases. Lung Cancer.

[23] Winter SF, Minna JD, Johnson BE, Takahashi T, Gazdar AF, Carbone DP (1992). Development of antibodies against p53 in lung cancer patients appears to be dependent on the type of p53 mutations. Cancer Res.

[24] Tsao MS, Aviel-Ronen S, Ding K, Lau D, Liu N, Sakurada A (2007). Prognostic and predictive importance of p53 and RAS for adjuvant chemotherapy in non-small cell lung cancer. J Clin Oncol.

[25] Groeger AM, Esposito V, De Luca A, Cassandro R, Tonini G, Ambrogi V (2004). Prognostic value of immunohistochemical expression of p53, bax, Bcl-2 and Bcl-x_L_ in resected non-small cell lung cancers. Histopathology.

[26] Huncharek M, Kupelnick B, Geschwind JF, Caubet JF (2000). Prognostic significance of p53 mutations in non-small cell lung cancer: a meta-analysis of 829 cases from eight published studies. Cancer Lett.

